# The Effect of Millisecond Pulsed Electric Fields (msPEF) on Intracellular Drug Transport with Negatively Charged Large Nanocarriers Made of Solid Lipid Nanoparticles (SLN): In Vitro Study

**DOI:** 10.1007/s00232-016-9906-1

**Published:** 2016-05-12

**Authors:** Julita Kulbacka, Agata Pucek, Kazimiera Anna Wilk, Magda Dubińska-Magiera, Joanna Rossowska, Marek Kulbacki, Małgorzata Kotulska

**Affiliations:** 1 Department of Medical Biochemistry, Medical University, Chałubińskiego 10 St, 50-368 Wroclaw, Poland; 2 Department of Organic and Pharmaceutical Technology, Faculty of Chemistry, Wrocław University of Science and Technology, Wybrzeże Wyspiańskiego 27, 50-370 Wrocław, Poland; 3 Department of General Zoology, Zoological Institute, University of Wroclaw, Sienkiewicza 21 St, 50-335 Wroclaw, Poland; 4 Institute of Immunology and Experimental Therapy Polish Academy of Sciences, Rudolfa Weigla 12, 53-114 Wroclaw, Poland; 5 Polish-Japanese Academy of Information Technology, Koszykowa 86, 02-008 Warsaw, Poland; 6 Department of Biomedical Engineering, Faculty of Fundamental Problems of Technology, Wroclaw University of Science and Technology, Wybrzeże Wyspiańskiego 27, 50-370 Wroclaw, Poland

**Keywords:** Millisecond pulsed electric field, Electroporation, Solid lipid nanocarriers, Coumarin-6, Drug delivery

## Abstract

Drug delivery technology is still a dynamically developing field of medicine. The main direction in nanotechnology research (nanocarriers, nanovehicles, etc.) is efficient drug delivery to target cells with simultaneous drug reduction concentration. However, nanotechnology trends in reducing the carrier sizes to several nanometers limit the volume of the loaded substance and may pose a danger of uncontrolled access into the cells. On the other hand, nanoparticles larger than 200 nm in diameter have difficulties to undergo rapid diffusional transport through cell membranes. The main advantage of large nanoparticles is higher drug encapsulation efficiency and the ability to deliver a wider array of drugs. Our present study contributes a new approach with large Tween 80 solid lipid nanoparticles SLN (i.e., hydrodynamic GM-SLN—glycerol monostearate, GM, as the lipid and ATO5-SLNs—glyceryl palmitostearate, ATO5, as the lipid) with diameters DH of 379.4 nm and 547 nm, respectively. They are used as drug carriers alone and in combination with electroporation (EP) induced by millisecond pulsed electric fields. We evaluate if EP can support the transport of large nanocarriers into cells. The study was performed with two cell lines: human colon adenocarcinoma LoVo and hamster ovarian fibroblastoid CHO-K1 with coumarin 6 (C6) as a fluorescent marker for encapsulation. The biological safety of the potential treatment procedure was evaluated with cell viability after their exposure to nanoparticles and EP. The EP efficacy was evaluated by FACS method. The impact on intracellular structure organization of cytoskeleton was visualized by CLSM method with alpha-actin and beta-tubulin. The obtained results indicate low cytotoxicity of both carrier types, free and loaded with C6. The evaluation of cytoskeleton proteins indicated no intracellular structure damage. The intracellular uptake and accumulation show that SLNs do not support transport of C6 coumarin. Only application of electroporation improved the transport of encapsulated and free C6 into both treated cell lines.

**Electronic supplementary material:**

The online version of this article (doi:10.1007/s00232-016-9906-1) contains supplementary material, which is available to authorized users.

## Introduction

One of the main objectives of nanomedicine technology is the delivery of poorly soluble drugs and/or supporting their bioavailability, improvement of drug targeting to the target cells with simultaneously reduced toxicity of normal cells, and additional control of the location and the rate of drug release. Currently, nanosized carriers up to 200 nm in diameter are selected for pharmaceutical and medical applications. Small sizes of nanoparticles increase the probability of their efficient transport into the cells. However, intracellular delivery system may benefit from various types of carriers, ranging in diameter from 30 nm to several micrometers. The main advantage of all nanocarriers is their ability to provide separate chemical environments, which can protect the loaded cargo from a potential damage (Lamch et al. 2014; Szczepanowicz et al. 2014; Lima et al. 2013; Puglia et al. 2014; Wissing et al. 2004; Bazylińska et al. 2012, 2014b). Nanosized carriers are currently regarded as one of the most promising directions in pharmaceutical research. They allow encapsulation of a complex cargo and its selective delivery to cells, enabled with a variety of carrier surface modifications (Lamch et al. 2014; Bazylińska et al. 2012, 2014a, b; Paliwal et al. 2014; Tran et al. 2014; de Morais et al. 2014; Saadeh et al. 2014). Large nanoparticles have many advantages but there is still a great challenge concerning their effective delivery into targeted cells. New approaches are being developed but new research in this field is still required. Lai et al. (2007) suggested additional coating of large nanoparticles to enable their rapid penetration into physiological human mucus. So far there is no method suitable for effective delivery of large carriers or vesicles, thus enhancement of their intracellular transport is needed. SLNs are considered the next generation of delivery system, after liposomes. Positively charged nanoparticles are advantageous in targeted drug delivery with regard to negatively charged cell surface. However, the research of liposomes showed that both positive and negative charges could enhance the delivery of liposomes to cells through adsorptive endocytosis and the extension of the half-life clearance of liposomes from the blood. The half-life clearance can take from a minute to hours and the distribution to organs can be controlled in part by changing the physical properties of nanoparticles, such as size, charge, and fluidity. However, some authors indicate that positively charged lipids are not approved by FDA for clinical use (Makholf et al. 2011). Zeta potential of liposomes or nanoparticles is an important factor for their cytotoxicity, and in some cases, cationic charge is demanded to increase the cytotoxicity (Liu et al.^2012^; Müller et al. 2001).

In this study, solid lipid nanoparticles (SLNs) were applied. SLNs are nanocarriers composed of a lipid which is solid both at body and room temperatures, stabilized by a suitable surfactant. Aqueous SLN dispersions are alternative colloidal systems to polymeric nanoparticles, liposomes, and emulsions, exhibiting many advantages, such as good stability, high drug payload, possibility of controlled drug release and drug targeting, low production costs (Mehnertet al. 2001). SLNs can be an alternative system to emulsions, liposomes, microparticles, and their polymeric derivatives. Lipid nanoparticles with a solid matrix have been proposed for encapsulation of many substances, such as chemotherapeutics (Wissing et al. 2004), fluorescent dyes (Calderón-Colón et al. 2015), photosensitizers (Lima et al. 2013), and chemical UV-filters (Puglia et al. 2014). SLNs are considered to be the next generation delivery system, after liposomes. Their most advantageous features include the solid state of the particle matrix, the ability to protect chemically unstable cargo against chemical decomposition, and the ability to modulate drug release (Műller et al. 2000).

The work reported here extends our recent studies on delivery of large nanocarriers with fluorescent cargo, their fabrication and imaging, drug encapsulation, release profiles, and biological impact (Szczepanowicz et al. 2014; Bazylińska et al. 2012; Bazylińska et al. 2014a, b; Lamch et al. 2014). This research is focused mainly on the effects of combination of large-type SLNs with electroporation (EP) on molecular transport of a loaded substance into normal and cancer cells. EP is induced with a pulsed millisecond electric field applied either before or after the addition of the nanocarriers into the solution with cells; in which the first mode enables an additional effect of electrophoresis. Such nanocarriers, combined with EP may lead to more effective transport of multidrug component and its enlarged dose to the target cells and simultaneous cargo protection.

## Materials and Methods

### Materials

**Fig. 1:**
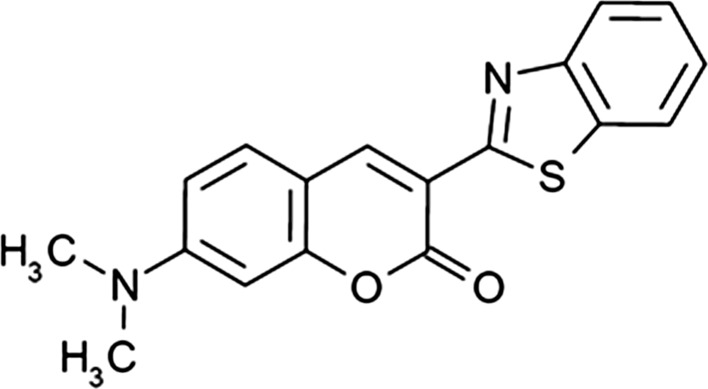
Molecular structure of coumarin 6 (C6)

Two biodegradable lipids were selected to prepare solid lipid nanoparticles: glycerol monostearate (GM) with the melting point at 58–59 °C and glyceryl palmitostearate (so called Precirol and denoted as ATO5) with the melting point at 56 °C. Both of them are recognized as Generally Recommended as Safe (GRAS) and have precedents of use in approved pharmaceutical products. Lipids for SLN production were supplied as follows: GM from CRODA Inc. and Precirol ATO5 from Gattefosse, S.A. (France). Tween 80 (T80), which was used as a non-ionic surfactant, tetrahydrofuran (THF), and coumarin 6 (C6; 3-(2′-benzothiazolyl)-7-diethylaminocoumarin; structure shown in Fig. 1) were purchased from Sigma-Aldrich. Water used for all experiments was doubly distilled and purified by means of a Millipore (Bedford, MA) Milli-Q purification system.

### Preparation of SLN Dispersions

SLN dispersion was obtained with the ultrasound technique, coupled with previous hot homogenization (Bazylińska et al. 2014a, b). The total amount of lipid phase (GM or ATO5) was kept constant in all lipid nanoparticle suspensions (4 %, w/w). The lipid nanoparticles in suspension were stabilized using 0.5 % (w/w) concentration of surfactant (T80). Coumarin 6 was used in a concentration of 1 % (w/w) with regard to the solid lipid matrix.

Coumarin 6 was dissolved in 0.5 ml of THF and added to lipids. The mixture was left for a night to completely evaporate organic solvent. The lipid with embedded drug was melted by heating it at 5 °C above the melting point of the lipid. The aqueous phase (surfactant dissolved in water) was heated to the same temperature as the lipid phase, then added to molten lipid phase and homogenized together (T25 basic Ultra Turrax, IKA, USA) for 10 min at 16,000 rpm. The coarse hot (o/w) emulsion thus obtained was sonicated for 15 min at the temperature of 70 °C. The obtained suspension was cooled down to room temperature in ice bath to obtain solid lipid nanoparticles (denoted as GM-SLN and ATO5-SLN, see Table 1).

**Table 1:** Characteristics of coumarin 6-loaded solid lipid nanoparticles

System	*D*_H_ (nm)	PdI	*ζ* (mV)	EE (%)
GM-SLN	379.4	0.225	−27.4	98.68
ATO5-SLN	547.0	0.304	−22.1	98.54

*D*_*H*_ hydrodynamic diameter, *PdI* polydispersity index, *ζ* zeta potential, *EE* encapsulation efficiency, *GM-SLN* SLNs with glycerol monostearate (GM) used as the lipid, *ATO5-SLN* SLNs with glyceryl palmitostearate (AT05) used as the lipid

### Particle Size and Zeta Potential

The size measurements of the studied lipid nanoparticles were performed with dynamic light scattering (DLS), using a Malvern Zetasizer Nano ZS (Malvern Instruments, UK). The detection angle zetasizer is 173° in optically homogeneous square polystyrene cells. DLS yields the hydrodynamic diameter (D_H_), which is an intensity weighted mean diameter of the bulk population, and the polydispersity index (PdI) as a measure of the width of the particle size distribution. The results are given as the average of 3 measurements, each with 10 runs.

Zeta potential (*ζ*-potential) of the SLNs was determined with the microelectrophoretic method, using a Malvern Zetasizer Nano ZS apparatus. The applied field intensity was 20 V/cm. Each value was obtained as the average of three subsequent runs of the instrument, each with at least 20 measurements. All the measurements were performed at 25 °C.

### Shape and Morphology

The shape and morphology of the dry nanoparticles were studied using scanning electron microscopy (SEM) and atomic force microscopy (AFM). SEM measurements were made using JEOL JSM-6610LV (Jeol, Japan) at operation voltage of 16 keV. Before measurements, SLN dispersions were mounted on metal stubs for 24 h, for drying.

AFM measurements were made using the Veeco NanoScope Dimension V AFM with an RT ESP Veeco tube scanner. The scanning speed was 0.5 Hz and a low-resonance-frequency pyramidal silicon cantilever (resonating at 250–331 kHz) was employed at a constant force of 20–80 N/m. Before observations, SLNs were allowed to adsorb on a freshly cleaved mica surface for 24 h by dipping it into the suspension. Then, the excess of substrate was removed by rinsing the mica plates in double distilled water for 1 min and drying at room temperature for 6 h.

### Determining Entrapment Efficiency

The percentage of incorporated coumarin 6 in SLNs (entrapment efficiency) was determined using UV–Vis spectroscopy, after centrifugation of the aqueous dispersion (isolation of the nanoparticles), using Eppendorf centrifuge 5804 (Netheler Hinz GmbH). The UV absorbance measurements were performed using a Metertech SP8001 spectrophotometer with a 1-cm pathlength thermostated quartz cell. The cargo concentration was calculated using the calibration plot. All the measurements were performed in triplicate.

We determined the difference between the mass of C6 entrapped in SLNs and the unloaded C6, which was measured in the supernatant of the resulting nanoparticle dispersion after the centrifugation. The entrapment efficiency (EE) could be calculated by the following Eq. 1:

1 $${\text{EE}}\, [\% ] = \frac{\text{total amount of coumarin 6 - amount of free coumarin 6}}{\text{total amount of coumarin 6}} \times 100.$$

### Differential Scanning Calorimetry (DSC)

Differential scanning calorimetry (DSC) was used to characterize the thermal characteristic, especially the lipid melting temperature. Experiments were performed with Setaram 32 CS (SETARAM, France) at the heating rate of 5 °C/min in the temperature range of 0–250 °C. Samples were weighed (5–10 mg) on standard 40 μl aluminum pans and then sealed. An empty sealed pan was used as a reference. DSC measurements were performed with the lyophilized SLN. The onset temperature and melting point (peak maximum) were calculated.

### Cell Culture

The studies were performed on human colon adenocarcinoma (LoVo) and hamster ovarian fibroblastoid (CHO-K1) cell lines. Both cell lines were purchased from ATCC (American Type Culture Collection). CHO-K1 cells can be applied as a model for transport studies with the pulsed electric field due to very low expression of endogenous ion channels (Gamper et al. 2005). The cell line was selected for the model study of drug transport. Ovarian fibroblasts were grown in Ham’s F-12 K (Kaighn’s modification) medium (Gibco, Poland) with addition of 10 % fetal bovine serum (FBS, HyClone, Poland) and antibiotics (streptomycin/penicillin; Sigma). LoVo cells were derived from metastatic tissue characterized as colorectal adenocarcinoma of Dukes’ type C, grade IV (acc. ATCC general information). This classification means that distant metastases were present, with the involvement of lymph nodes. Colon adenocarcinoma cell line was grown in Ham’s F-12 medium (Gibco, Poland) with addition of 10 % fetal bovine serum (FBS, HyClone, Poland) and supplemented with antibiotics (penicillin/streptomycin; Sigma). The cell lines were cultured in plastic flasks of 25 or 75 cm^2^ (Nunc, Denmark), which were stored at 37 °C and 5 % CO_2_ in an incubator (Steri-Cult, Thermo Scientific, Alab, Poland). For the experiments the cells were detached by trypsinization (Trypsin 0.025 %; Sigma) and neutralized by the cell culture medium.

### Electroporation Protocol

Electroporation, applied on cells alone and with addition of free or encapsulated Coumarin-6 (EP + C6), was performed using ECM 830 Square Wave Electroporation System (BTX Harvard Apparatus, purchased from Syngen Biotech, Poland). After trypsinization and centrifugation (5 min, 1000 rpm) cells were counted ([cells density] = 3 × 10^6^/ml) and resuspended in 200 µl of EP buffer with low electrical conductivity of 0.12 S/m (10 mM KH_2_PO_4_/K_2_HPO_4_, 1 mM MgCl_2_, 250 mM sucrose, pH 7.4) (Weżgowiec et al. 2013). In case of C6 incubation, cells were suspended in EP buffer with free C6, [C6] = 5 μM, C6 encapsulated in ATO5-SLN and GM-SLN-based nanocarriers. Additionally, EP was performed with empty nanocarriers. SLN concentration was adjusted to C6 concentration. EP experiments were performed in two modes: (1) cells were electroporated simultaneously with SLNs; (2) SLNs were added to the cells after EP (delay up to 30 s). In each case, the same volumes of EP buffer and SLN suspension were maintained (ratio 1:1). Cell suspension was pulsed in a cuvette with two aluminum plate electrodes, applying electrical fields E(appl)=100 V/cm; 500 V/cm, or 1000 V/cm (Eq. 2):

2 $${\text{E(appl)}} = \frac{\text{U(appl)}}{\text{d\_electrode}}$$

*U*(appl) = 40, 200 or 400 V; (electrical field), d_electrode = 0.4 cm; (distance between electrodes in cuvette).

Five rectangular electrical pulses were delivered by electroporator ECM 830 (BTX, Syngen Biotech Poland), pulse duration of 1.5 ms and pulse train repetition frequency of 1 Hz. Control cells were not permeabilized, only incubated with EP buffer or with C6 in EP buffer. After pulsation, before further experiments, the cells were left for 10 min at 37 °C, centrifuged, resuspended in cell culture medium, and proceeded for viability assay and CLSM study for cells electroporated simultaneously with SLNs. Cells in which SLNs were added after the EP process were subsequently assessed by fluorescent microscopy and FACS analysis.

### FACS Analysis: PI and C6 Uptake

Flow cytometry analysis was performed for assessment of the cell ability to internalize propidium iodide and encapsulated coumarin 6 into the CHO-K1 and LoVo cells. Cells were detached with Trypsin–EDTA and subjected to EP protocol described in Sect. 2.8. Immediately before EP, propidium iodide (PI, P4170, Sigma) was applied to the cell suspension. Concentration of PI in cuvette for the EP buffer was [PI] = 20 μM. After EP process, the solution of SLNs with C6 was added to cell suspension. The concentration of C6 in the final volume was [C6] = 5 μM (200 µl of cells suspension + 200 µl of SLNs suspension). Then the cells were incubated for 10 min at 37 °C in a humidified atmosphere containing 5 % CO_2_. Then cells were washed in PBS and resuspended in 0.5 ml of PBS. Flow cytometric analysis was performed on a FACSCalibur flow cytometer (Becton–Dickinson). The fluorescence of PI was measured with FL-1 detector, the fluorescent signal for coumarin 6 was detected with FL-3 detector. Data analysis was performed with CellQuest software (Becton–Dickinson).

### The Viability Assay (MTT)

The cellular mitochondrial activity was determined with the MTT (3-(4,5-dimethylthiazol-2-yl)-2,5-diphenyltetrazolium bromide) assay. MTT assay was performed 24 h after the end of experiments to evaluate cells mitochondrial dehydrogenase activity (NADH) as a viability marker. The cells viability assay was performed according to the manufacturer’s protocol (In Vitro Toxicology Assay, Sigma). The absorbance was measured at 570 nm using multiwell plate reader (EnSpire Multimode Reader, Perkin Elmer, Poland). Three samples per each experiment were prepared. Additionally, each experiment was performed in 3 independent repetitions. Mean values and standard deviations of all results were calculated. The final results were expressed as the percentage of mitochondrial function relative to untreated control cells. The results obtained from viability assays were subjected to statistical analysis. The significance of differences between the mean values of different groups of cells was assessed with T-test, which returns the probability associated with the Students *t* test, carried out for each experiment individually and *n* ≥ 6. In the statistical estimations, the level of *P* ≤ 0.05 (*) was regarded as statistically significant.

### Fluorescent Microscopy (FL) for the Evaluation of C6 Uptake and Distribution

CHO-K1 and LoVo cells were prepared for fluorescent studies. Cells were primarily harvested on cover slides and different EP protocols were performed (EP alone, EP with C6, and EP with encapsulated C6) using a Petri-dish electrode. EP process was performed in the presence of propidium iodide (20 µM in EP buffer as in the Sect. 2.8) to evaluate EP efficiency. Then SLNs suspensions with C6 (5 µM) were added to the slides. Cells were incubated for 10 min in 37 °C, then washed with PBS, fixed with 4 % paraformaldehyde, and washed with PBS. Then, the slides were mounted with Roth fluorescent mounting medium containing DAPI (4,6-diamidino-2-phenylindole) for nuclei staining. C6 in cells was observed using excitation wavelength *λ*_exc_ = 485 nm and observed in *λ*_em_ = 503 nm; DAPI with *λ*_exc_ = 350 nm and *λ*_em_ = 470 nm, PI with *λ*_exc_ = 536 nm and *λ*_em_ = 617 nm. For imaging, Olympus BX53 fluorescent microscope with X-Cite 120PC Q adapter was used.

### CLMS for the Evaluation of Cytoskeleton and Intracellular C6 Distribution

CHO-K1 and LoVo cells were prepared for immunofluorescence. For the observation 10 min after EP, cells were primarily harvested on cover slides and EP protocols were performed with the Petri pulser electrode and then cells were fixed with 4 % paraformaldehyde (PFA) in PBS. For visualization, 24 h after experiments (EP, EP with C6, and EP with encapsulated C6) cells were grown on coverslips for 24 h, then fixed with 4 % PFA in PBS, and permeabilized with 0.5 % Triton X-100 in PBS (v/v) for 5 min. at room temperature. Then, they were blocked with 1 % Bovine serum Albumin (BSA) in PBS (for 30 min. at room temperature). PBS was used for all washing steps. The following antibodies were used: primary antibody—monoclonal anti-β-tubulin antibody produced in mouse (overnight incubation at 4 °C; 1:100; Sigma-Aldrich); secondary antibody—donkey anti-mouse IgG Cy5 conjugated (for 60 min. at room temperature; 1:50; Jackson ImmunoResearch). For F-actin identification, Alexa 546-conjugated phalloidin was used (at a concentration of 1 nM; Life Technologies). DNA was stained with DAPI (Life Technologies, 4,6-diamidino-2-phenylindole; 0.57 µM). Cell were mounted in fluorescence mounting medium (DAKO). C6 in cells was excited with wavelength *λ*_exc_ = 473 nm and detected with *λ*_em_ = 650 nm. For imaging, Olympus FluoView FV1000 confocal laser scanning microscope (Olympus) was used. The images were recorded with Plan-Apochromat 60x oil-immersion objective. Additionally, images were analyzed with ImageJ software (Interactive 3D Surface Plot). A two-dimensional graph of the pixels intensities (fluorescence intensity) was analyzed along a marked line, within a selected single cell in the image. The line was performed on the selected single cell from each CLSM picture.

## Results

In our study, coumarin 6-loaded SLN dispersions were fabricated by the ultrasound technique coupled with the previous hot homogenization. This technique is widespread, offers low cost, and low energy consumption, as well as easy handling (Wissing et al. 2004; Fathi et al. 2013).

### Characterization of the C6-Loaded Solid Lipid Nanoparticles

**Fig. 2:**
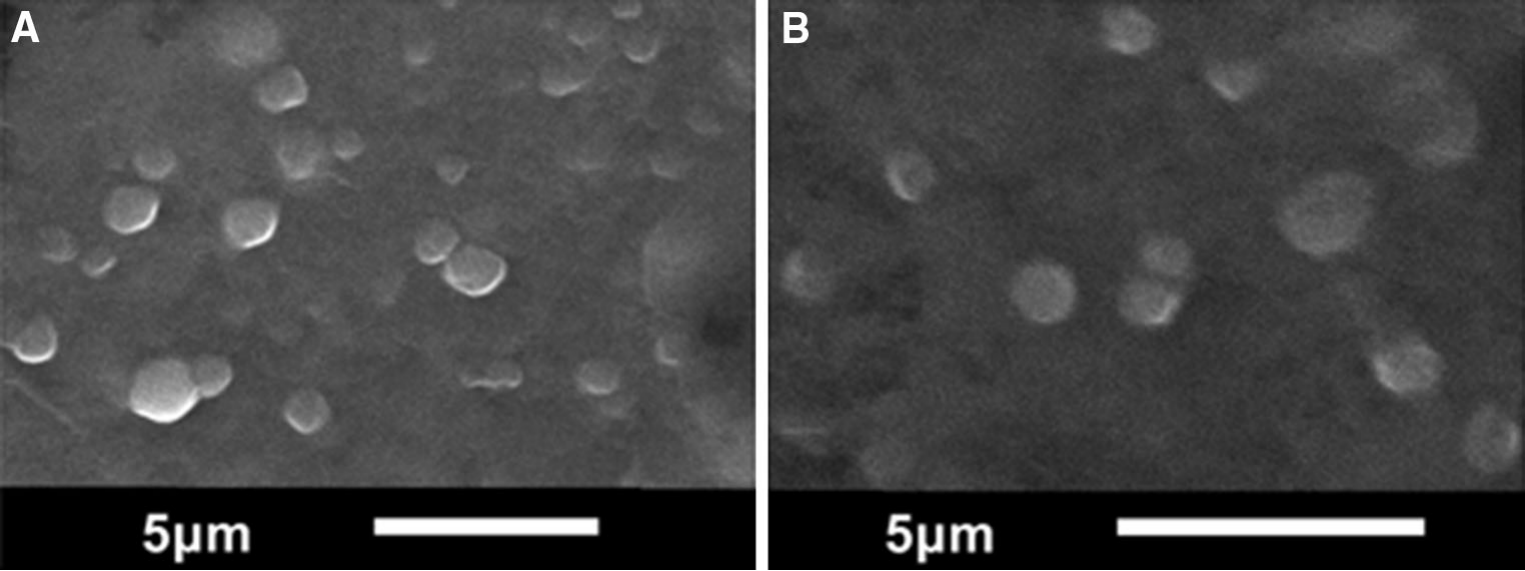
SEM images of **a** GM-SLN, **b** ATO5-SLN

In Table 1, presented are values of hydrodynamic diameter (D_H_), polydispersity index (PdI), hydrophobic cargo entrapment efficiency (EE), and zeta potential (ζ) of the obtained samples GM-SLN and ATO5-SLN. Accordingly, it can be concluded that the SLN dispersions displayed an average size of 380 nm of diameter for GM used as a lipid, and 550 nm—for ATO5. Values of PdI were ca. 0.225 and 0.304 nm, respectively, proving quite monodisperse nanoproducts. Lipid nanoparticles were negatively charged on the surface, exhibiting the **ζ** potential above |20| mV, which may suggest a reasonable stability of the nanoparticles’ dispersions. Furthermore, the SEM and AFM images of GM-SLN and ATO5-SLN (see Fig. 2) provided the sizes of samples similar to the values obtained primarily by DLS and indicated that the nanostructures assumed semi-spherical shapes and they did not show any enhanced aggregation.

**Fig. 3:**
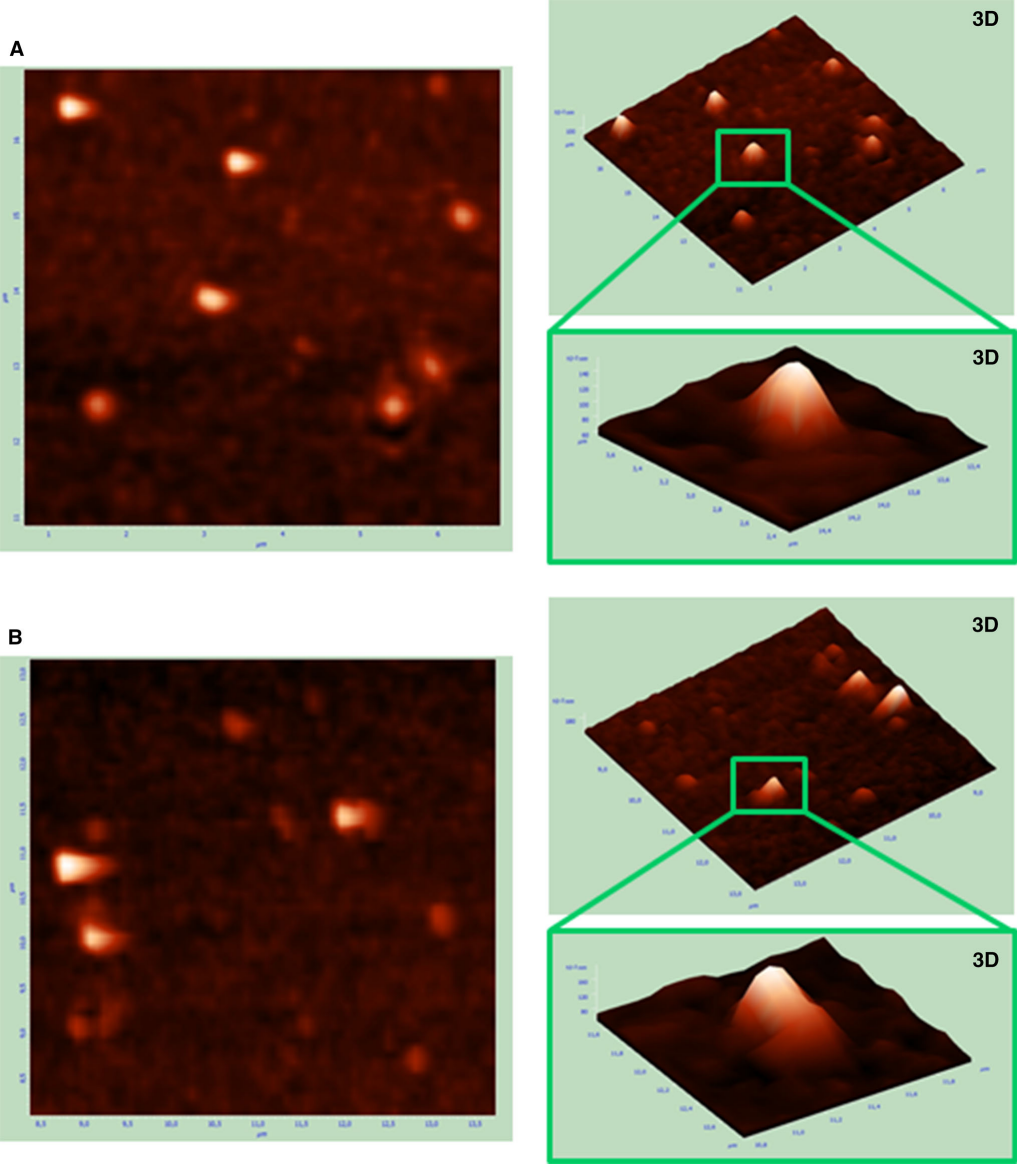
AFM images of **a** GM-SLN, **b** ATO5-SLN

**Fig. 4:**
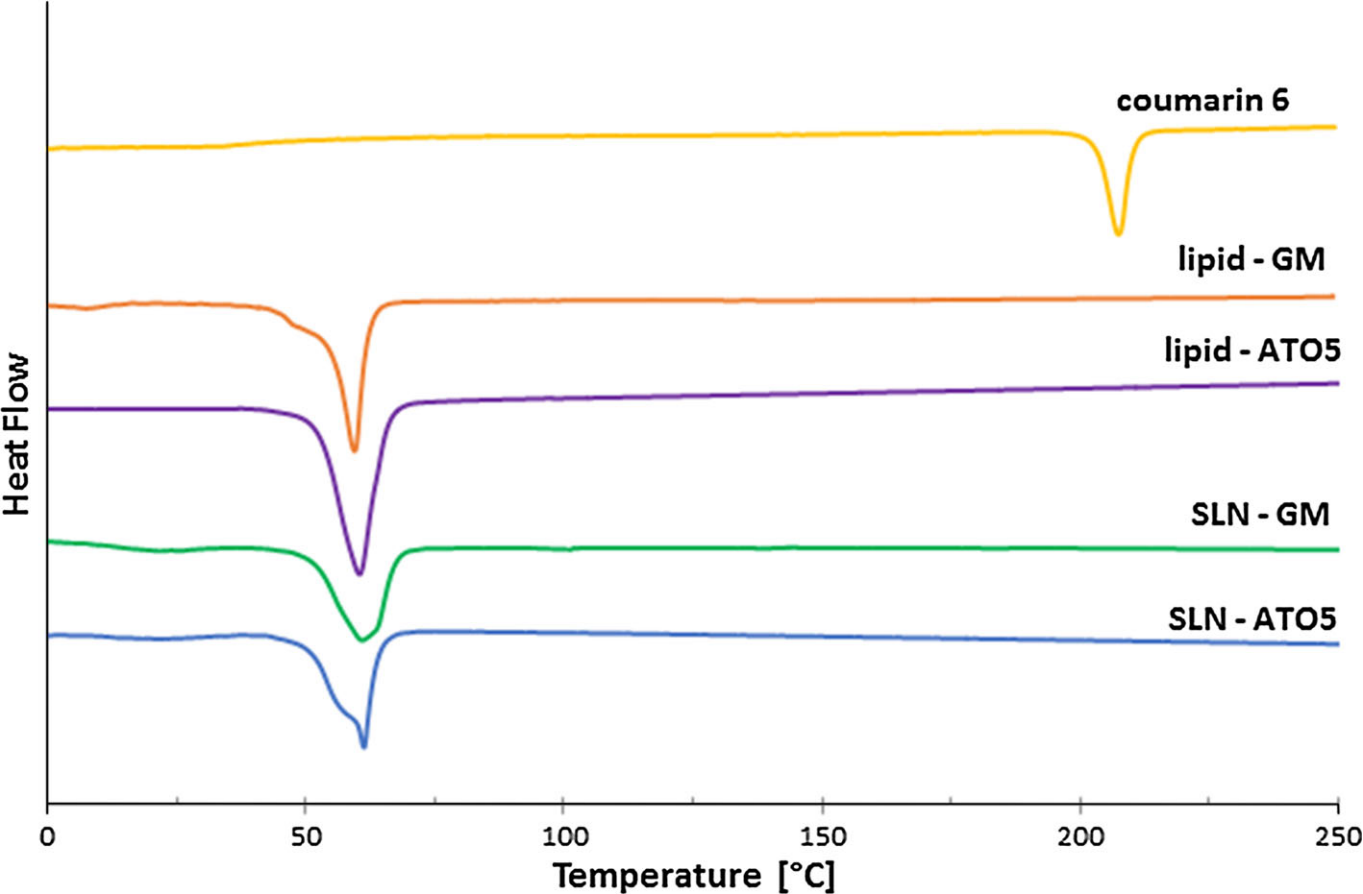
DSC profiles of the melting process of C6, lipids, and C6- loaded SLNs

The DSC thermograms of C6, pure lipids, and coumarin-loaded SLN powder are shown in Fig. 3. The studied lipids—ATO5 and GM—showed well-defined endothermic peaks, indicating pure crystalline structure. C6 showed an exothermic sharp peak at the melting point of 207.44 °C, responsible for the crystalline nature of the cargo. The peaks from the nanoparticles are broader and shallower than those of pure lipids, which indicates their less ordered crystalline structure. However, both systems showed a single peak, which proved that C6 was completely encapsulated inside the lipid matrix (Fathi et al. 2013). The melting point of ATO5 is 57.57 °C, but when this lipid is used for the preparation of nanoparticles this value increases to 61.42 °C, which is the result of the cargo (Casadei et al. 2006). For the system with GM, used as the lipid, these values are 59.46 and 61.10 °C, respectively. Lyophilized SLNs display a offset of the melting temperature to higher temperatures as compared to that of the pure lipid. Endothermic peaks of pure lipids correspond to their melting points (Fig. 4).

### Viability Assay

**Fig. 5:**
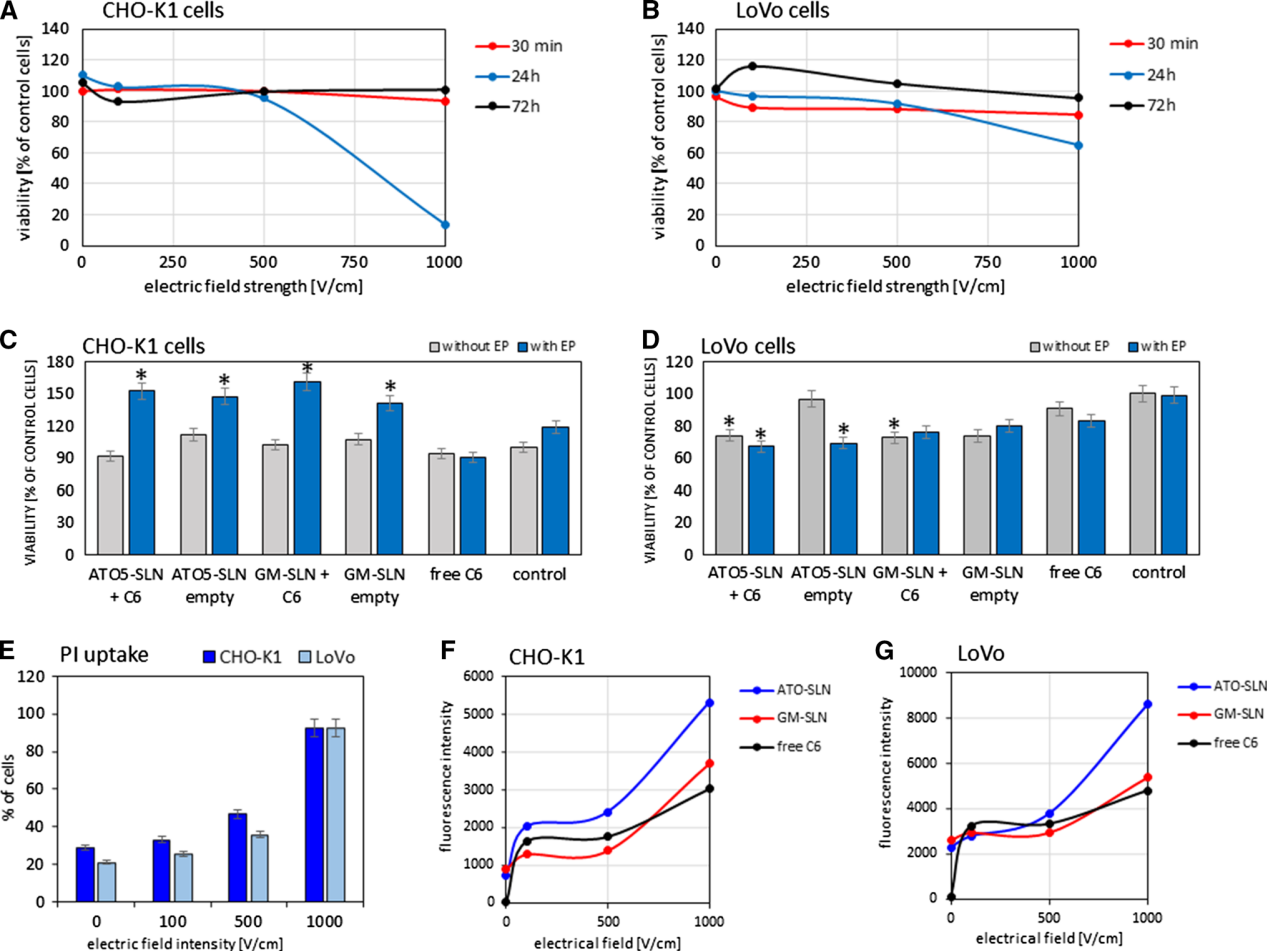
*Top row* the effect of EP parameters on cell viability (MTT test): **a** CHO-K1 cells; **b** LoVo cells. *Middle row* the viability of cells after treatment with free C6, empty, and C6-loaded nanoparticles (GM-SLN and ATO5-SLN); SLNs and free C6 added before EP: **c** CHO-K1 cells, **d** LoVo cells. *Bottom row* FACS analysis, uptake of PI and free C6, added before EP, and SLNs added post-EP: **e** CHO-K1 and LoVo cells—dependency of PI uptake (percent of cells) on EP parameters; **f** CHO-K1 cells—uptake of free and encapsulated C6 (fluorescence intensity); **g** LoVo cells—uptake of free and encapsulated C6 (fluorescence intensity)

The effect of EP parameters on both cell lines is presented in Fig. 5a for CHO-K1 cells and in Fig. 5b for LoVo cells. The most cytotoxic effect was observed after 24 h for EP at 1000 V/cm, for both cell lines but with stronger effect on CHO-K1 cells. The influence of free SLNs, SLNs with encapsulated coumarin 6, and EP—alone and in combination with empty or loaded SLNs is presented in Fig. 5c for CHO-K1 and Fig. 5d for LoVo cells. All viability results were obtained for experiments performed with pre-addition of free C6 and encapsulated in SLNs. The C6 concentration of 5 μM, applied in our experiments, is regarded as non-cytotoxic and can be used as a fluorescent marker. Higher concentrations would induce the cytotoxic effect leading to significant cell destruction or even cell death (Chuang et al. 2007; Ma et al. 2012). The EP parameters (500 V/cm; 5 imp. each of 1.5 ms duration) for SLNs delivery were selected as optimal and non-toxic according to our MTT results and the available literature reports (Čemažar et al. 2002; Yu et al. 2011; Zou et al. 2014; Huang et al. 2014). Our results confirm the published data and indicate that no significant cytotoxic effect was observed in the case of both cell lines. The stimulative impact on proliferation was observed for the CHO-K1 cells when pulsed electric field was applied in the presence of the solid lipid nanocarriers (Fig. 5a). Then, an increase of mitochondrial activity was observed at the level of 50 %. However, in LoVo cells free C6, either with EP or without it, induced only ca. 10 % decrease of cellular viability. In the presence of ATO5-SLN and GM-SLN nanoparticles, even ca. 20–30 % decrease of cellular viability was observed. Empty ATO5-SLN, which were not accompanied by the pulsed electric field, maintained cell viability at the control level.

### PI and C6 Uptake: FACS Analysis

The fluocytometric analysis is presented in Fig. 5e–g. The results for PI uptake are presented for experiments performed with pre-addition of PI or C6, immediately before electroporation. However, the results for C6 in SLNs are presented for experiments in which nanoparticles were added after EP. The dot plots indicate quantitative distribution of cells with PI and C6 (Fig.SI-1). The study of EP parameters indicated that cells of both lines could effectively incorporate PI after EP, particularly after EP at 500 and 1000 V/cm (Fig. 5e). The evaluation of free and encapsulated C6 uptake by cells showed the increase of fluorescent signal after EP at 100 V/cm in both cell lines, but the highest fluorescent signal was observed for cells treated with 1000 V/cm and C6 in ATO5-SLNs (Fig. 5f, g).

### Coumarin-6 Intracellular Distribution

**Fig. 6:**
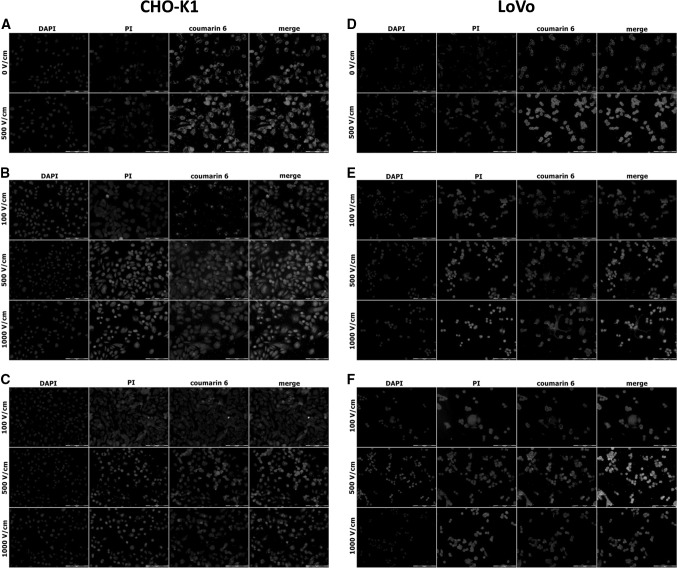
The fluorescence microscopy analysis of C6 and PI uptake together with DAPI staining indicating cells nuclei. *Top row* free C6 uptake (without EP and EP at 500 V/cm, C6 added before EP): **a** CHO-K1 cells; **b** LoVo cells; *Middle row* C6-loaded into GM-SLNs, nanoparticles added immediately post-EP (100, 500 and 1000 V/cm): **c** CHO-K1 cells; **d** LoVo cells; *Bottom row* C6-loaded into ATO5-SLNs, nanoparticles added immediately post-EP (100, 500 and 1000 V/cm): **e** CHO-K1 cells; **f** LoVo cells

The intracellular distribution determined by fluorescent microscopy of free C6, encapsulated and combined with EP in ovarian fibroblasts and colon adenocarcinoma cells is presented in Fig. 6a–f. These results concern experiments in which loaded nanoparticles or free C6 were added immediately after EP. In both cell lines, we could observe the highest number of cells stained with propidium iodide after EP at 500 and 1000 V/cm. We could also observe that for C6 encapsulated in SLNs (ATO5 and GM), the most intense fluorescent signal was also induced at these parameters. C6 in SLNs was distributed in the cytoplasm and in some cases it could be observed that it was clinging to cell membranes (Fig. 6c for CHO-K1 cells but in particular in LoVo cells Fig. 6e). Also in control cells treated with free C6 (Fig. 6a, d), we could observe enhanced fluorescent signal after EP (500 V/cm). Our observations indicated similar fluorescent signal of encapsulated C6 in both cell lines, in particular, after EP with the electric field at 500 V/cm. In both cases, with GM-SLN and ATO5-SLN, we could observe the enhanced transport, a little less efficient as in case of free C6.

**Fig. 7:**
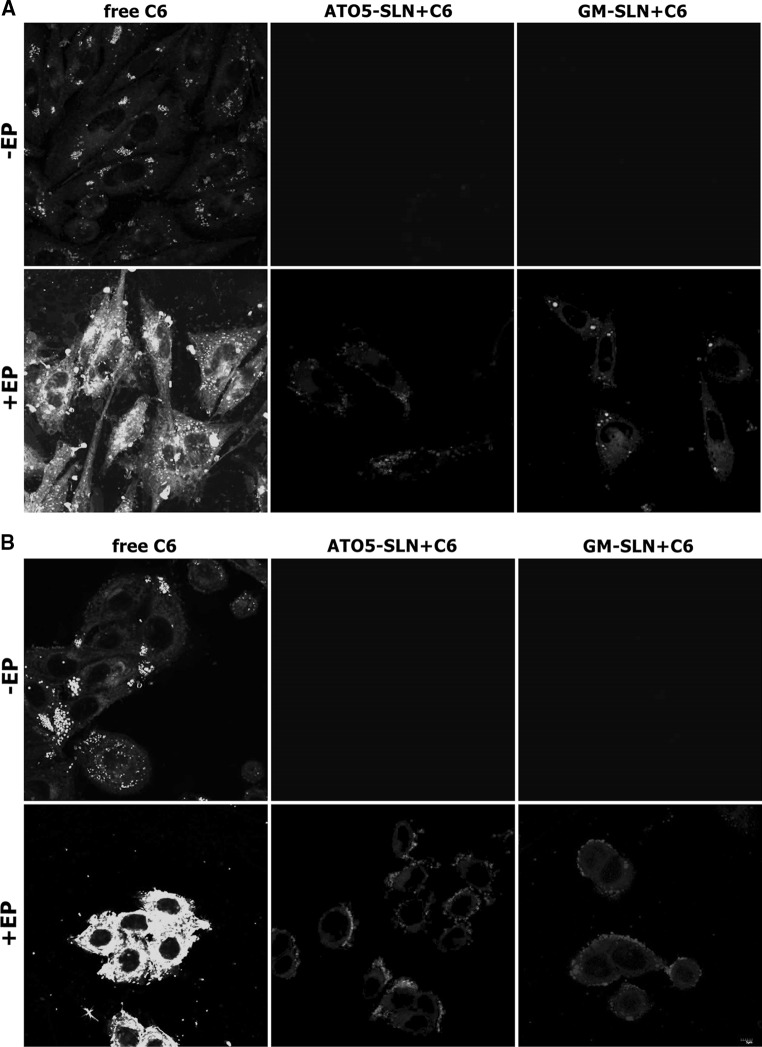

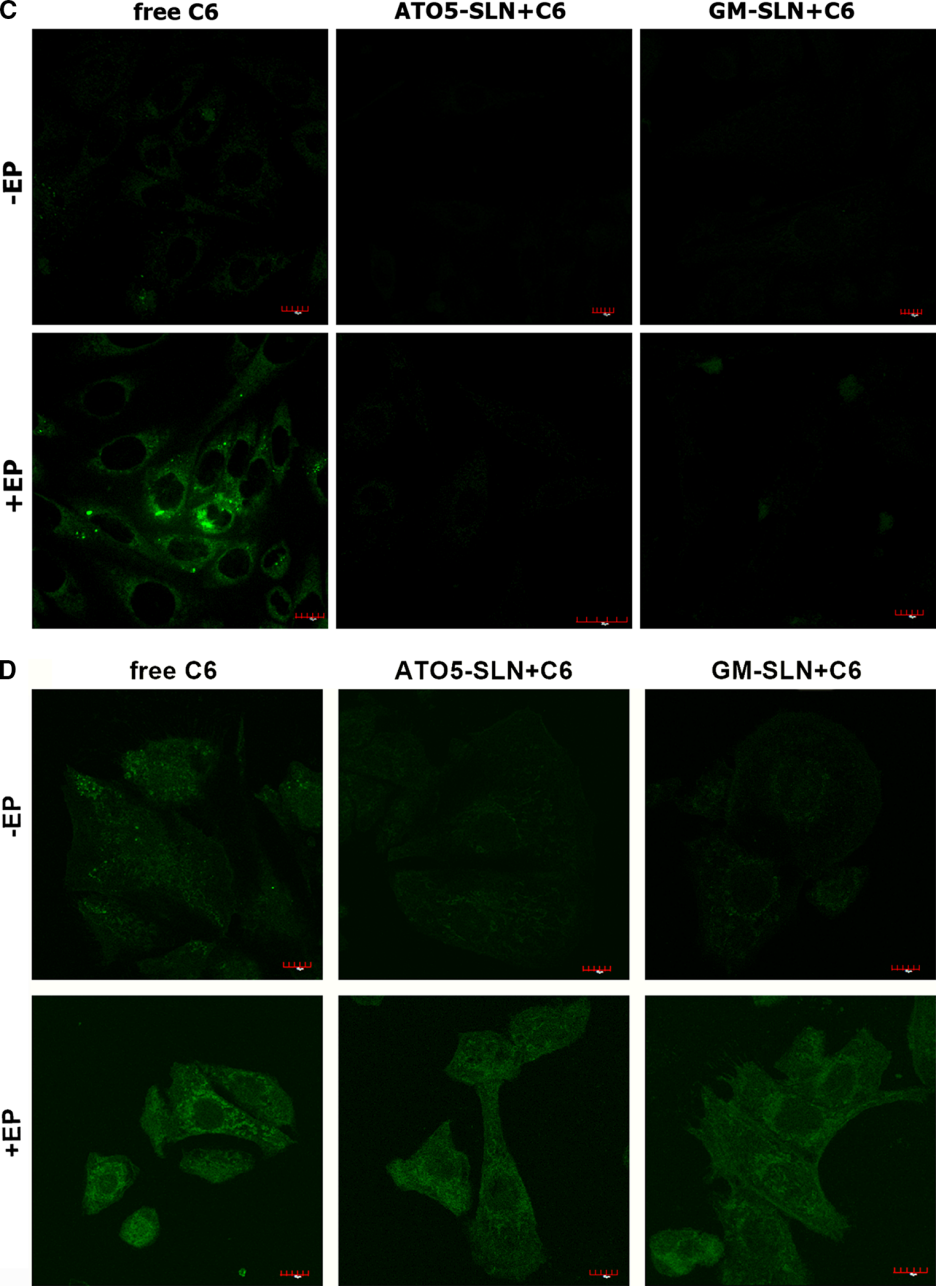
The evaluation C6 distribution in the cells using CLSM analysis. C6-loaded in nanoparticles (GM-SLN and ATO5-SLN) added before EP at 500 V/cm (5 pulses, 1.5 ms each) or no electroporation applied. Images obtained 10 min after the procedure: **a** CHO-K1 cells and **b** LoVo cells; and images obtained 24 h after the procedure: **c** CHO-K1 cells and **d** LoVo cells

**Fig. 8:**
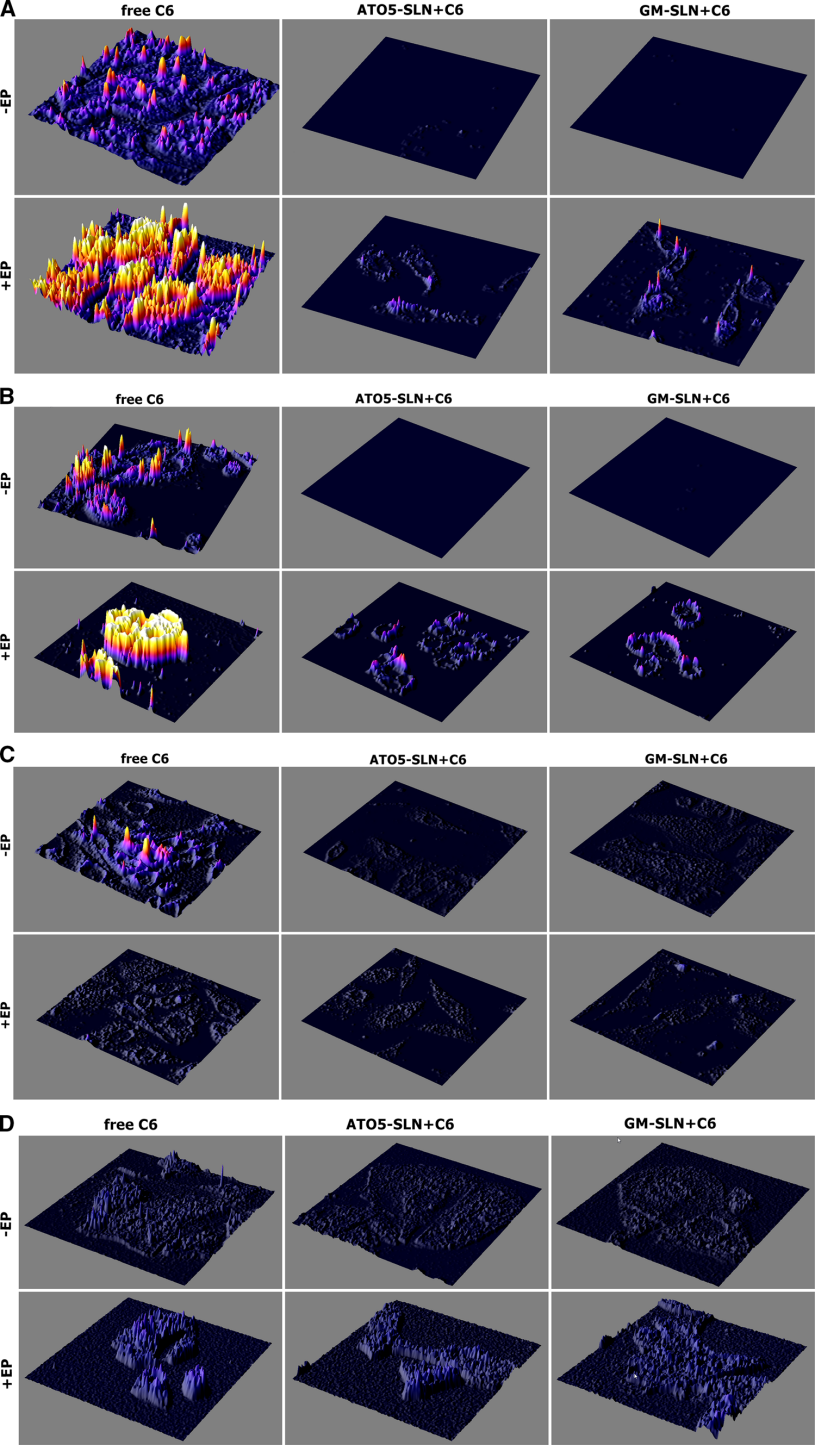
The analysis of fluorescence intensity from CLSM analysis. C6-loaded nanoparticles (GM-SLN and ATO5-SLN) added before EP at 500 V/cm (5 pulses, 1.5 ms each) or no electroporation applied. Images obtained 10 min after the procedure: **a** CHO-K1 cells and **b** LoVo cells; and images obtained 24 h after the procedure: **c** CHO-K1 cells and in **d** LoVo cells

The results showing intracellular distribution of C6 determined by CLSM after experiments, where nanoparticles were added before EP are presented in Fig. 7a, b 10 min after treatment incubation, and in Fig. 7c, d 24 h after treatment. Additional analysis of fluorescence intensity was performed with Fiji software (Image J) and presented in Fig. 8a, b from samples fixed 10 min after experiment and in Fig. 8c, d from samples fixed 24 h after experiment.

The results obtained for CHO-K1 cells indicate that only free C6 can be efficiently transported by EP inside the cells (Figs. 7a, c, 8a, c). The fluorescent signal for C6 in ATO5 or GM nanoparticles remained at the level of the cells autofluorescence. Only in the case of electroporated GM + C6 green fluorescence was observed in single points (Fig. 7a). In case of LoVo cells, we could detect the same weak level of fluorescence in non-electroporated cells. After EP, we could observe the increased accumulation of free C6, but it was weaker than in the case of CHO-K1. After EP with C6 encapsulated in large ATO5 and GM nanoparticles, we could observe almost the same level of fluorescence as in the case of free fluorescent marker (Fig. 7b). These data are more evident in Fig. 7b, where histograms present the fluorescent intensity in selected cell. Our results indicate that SLNs alone are not delivered into cells. Only after electroporation the intracellular transport of SLNs could be detected.

### Cytoskeleton Evaluation: CLSM Study

The visualization of cytoskeleton proteins in ovarian fibroblasts and colon adenocarcinoma cells is presented in Fig.SI-2 A and B (electroporated) for CHO-K1 cells and in Fig.SI-3 A and B for LoVo cells. The cytoskeleton visualization, for C6 free and encapsulated in SLNs, is presented for experiments in which nanoparticles were added before EP. The evaluation of actin and tubulin indicated that normal cytoskeleton structure was observed in both treated cell lines. Only in the case of electroporated CHO-K1 cells with GM + C6 (Fig.SI-2B) we could observe some tubulin reorganization, in the form of small fragments distributed through intercellular environment. The short time of incubation (10 min) with free and encapsulated C6 did not affect shape and morphology of treated cells.

## Discussion

The electropermeabilization technology can be effectively applied in transport of molecules, such as chemotherapeutics in electrochemotherapy (Mir et al. 1992, 2003, 2014; Sersa et al. 1998, 2008, 2009; Heller et al. 2002; Čemažar et al. 1998a, 1998b; Kulbacka et al. 2014; Saczko et al. 2014; Vasquez et al. 2014; Campana et al. 2014; Yarmush et al. 2014; Cadossi et al. 2014; Miklavcic et al. 2014) or nucleic acids in gene therapy (Jaroszeski et al. 1995, 1999, 2000, 2004; Heller et al. 1996, 2001, 2005, 2010; Ugen and Heller 2003; Gillbert et al. 2002; Aihara et al. 1998; Miyazaki and Aihara 2002; Rols et al. 1998; Gibot and Rols 2015; Faurie et al. 2003; Orio et al. 2013; Forde et al. 2014; Emr et al. 2014; Kalli et al. 2014; Gehl 2014; Takei 2014; Hu and Li 2014). In the case of electrochemotherapy, electropores appearing in the cell membrane after application of the electric field enable transport of small non-lipophilic drug molecules into the cell. Larger molecules, such as nucleic acids, enter the cell due to the cell membrane electropermeabilization, which results from EP and more widespread lipid disorganization. Their transport also needs the electrophoretic effect of the electric field, possible due to the electrical charge of nucleic acid particles (Kee et al. 2011; Mir 2014). Some authors have already applied EP to support nanoparticles transport. Ramos et al. (2002) applied EP for facilitated delivery of the large lipid unilamellar vesicles (LUVs). The authors presented that LUVs may undergo fusion with electropermeabilized cells. Additionally, it was observed that the uptake of LUVs increased with the number of pulses and the amount of liposomes that were added to the cell suspension. Henri et al. (2015) also experimented with LUVs. It was shown that when LUVs were electrostatically carried into contact with electropermeabilized cells by a salt bridge, their content was delivered into the cytoplasm of the electropermeabilized cells. The authors also proved that pulsing cells in the presence of highly fusogenic empty LUVs improved the viability of cells. Yu et al. (2011) indicated that EP assisted metallic nanoparticle delivery. The authors concluded that the silver nanoparticle delivery rate was the most efficient with the EP parameters slightly higher than those used in our research, i.e., 4 consecutive rectangular pulses at 875 V/cm, 1 ms and 10 ms durations. It was also indicated that low temperature (0–4 °C) was necessary for improving the delivery efficiency of silver nanoparticles. Similar parameters that were applied in the present study were also used in the case of gold nanoparticles; Huang et al. (2014) applied gold nanoparticles enhanced by 625 V/cm (1 pulse) for polyplex delivery to mammalian cells. Čemažar et al. (2002) demonstrated that electric pulses of lower amplitude and longer duration (600 V/cm, 5 ms) generated significantly better transfection efficiency of plasmid DNA in liposomes, in comparison to electric pulses of higher amplitude and shorter duration (1200 V/cm, 0.1 ms). Zu et al. (2014) subjected a highly conductive solution of gold nanoparticles (AuNPs) to electroporation. Using EP (475 V/cm and 10 ms) they demonstrated the enhanced DNA delivery with simultaneously increased cellular viability of mammalian cells. The results obtained by Kim et al. (2011) showed that, compared to cationic nanoparticles, anionic nanoparticles had a better potential as electro-mediated gene transporters at a low gene concentration. The approach involving electropermeabilization and nanoparticles delivery is still novel. As it was proved by Colacino et al. (2014), not only enhanced drug delivery may be expected from this new method but additionally the increased cancer cell cytotoxicity. The authors demonstrated the potential application of folic acid-conjugated cellulose nanocrystals in the potentiation of irreversible EP-induced cell death in folate receptor (FR)-positive cancers. Pre-incubation with FA-conjugated nanostructures (CNC-FA) led to a significant increase in cytotoxicity when followed by irreversible EP.

Negatively charged SLNs, applied alone, are not effective in drug delivery. Our study presents the potential of applying EP to enhance the transport of drugs encapsulated in large SLNs. In the study, we used two different cell lines—ovarian fibroblast CHO-K1 cells with very low expression of endogenous ion channels, and colon adenocarcinoma LoVo cells. Both types of the applied SLNs were relatively large (ca. 380 nm and 550 nm in diameter) and negatively charged. The results obtained from C6 uptake (FACS) and distribution (fluorescent microscopy) indicated that the external electrical field effectively supported transport of C6 encapsulated in SLNs. The enhanced drug delivery was observed when SLNs were added directly after EP into the cell culture. The enhanced transport with no cytotoxic effects was obtained with five 1.5 ms rectangular pulses and 500 V/cm in cancer and normal cell lines. The observed enhancement in SLNs transport into the LoVo cells, as an effect of electroporation, indicates its potential anticancer applications. Similar EP parameters (typically between 50 and 500 V/cm with millisecond pulses) occurred effective with regard to another barrier, such as a tissue (Davalos et al. 2005). Additionally, some studies based on computer modeling and simulations clearly showed that the effects of nanosecond, megavolt-per-meter pulsed electric fields and of low-voltage, millisecond pulses are very similar at the membrane level (Delemotte et al. 2012). Our experiments performed on cells electroporated with SLNs indicated the stimulative effect on cellular viability, i.e., the increased activity of mitochondrial dehydrogenase in particular in CHO-K1, which was measured by MTT assay.

Some authors observed fusion or electrophoretic effects in case of nanocarriers combined with electroporation applied after the addition of nanocarriers into the cell environment (Ramos et al. 2002; Henri et al. 2015). In our study, cancer LoVo cells showed amplified fluorescent signal in cellular cytoplasm after electroporation with SLNs. This effect was most probably due to the cell membrane electroporation, strengthened with the electrophoretic effect. However, this effect was not as potent as it was expected. The electrophoretic contribution of electric field in the experiments with pre-EP addition of SLNs appeared inefficient in drug delivery in normal cells. It was well visualized in CLSM study, where we could observe adhesive bounding of SLNs into external CHO-K1 membranes. However, our results raise the question on the mechanisms underlying these observations. Due to the large size of the carriers, application of the electric field must have enhanced the electrophoretic effect on the negatively charged carriers. This was observed in LoVo cells. However, EP did not enhance the SLNs transport into CHO-K1 cells. These varying sensitivities and cellular responses may be due to different characteristics of the cells. CHO-K1 cells have low expression of ion channels. Additionally, some studies report that CHO-K1 cells do not contain cytochrome P450 enzymes and cannot metabolize chemicals in culture (Born et al. 2000; McGregor et al. 1991). Other studies have shown that external electric fields could induce fusion of a wide variety of cell and artificial membranes (Dimitrov et al. 1995). This fusion effect was also observed in our studies on SLNs and it could have additionally increased the internalization of C6 in the cancer cells. Table 2 presents the main effects obtained with SLNs added either pre-EP or post-EP. As it was observed, SLNs without EP were not able to get into the cells. Our results show that electroporation performed on suspension of cells with SLNs leads to unsealing of SLNs membranes and final premature cargo release form nanocarriers, which is unfavorable in drug delivery process. EP performed before SLNs addition only causes permeabilization of the cellular membranes and enables safe delivery of the nanocarriers with their full cargo. According to the presented data, we can conclude that in case of large anionic SLNs post-electroporation addition of the nanocarriers is optimal for efficient drug delivery.

**Table 2:** The summary of the observed effects on large, anionic SLNs in combination with electroporation

SLNs added post-EP	SLNs added before EP	SLNs without EP
Enhanced transport of SLNs with active cargo	Fusion of SLNs and cells	No drug delivery
Permeabilization of cellular membranes only	Electrophoretic effect on anionic SLNs	
	Adhesive bounding of SLNs to external cell membranes	
	Unsealing of SLNs and premature release of cargo	

*SLN* solid lipid nanoparticles, *EP* electroporation

Our results demonstrated that the application of SLNs with the pulsed millisecond electric fields could enhance intracellular transport of large nanoparticles into cancer cells, such as LoVo, and it does not affect cells viability if the carrier content is not cytotoxic. Significant improvement in drug uptake can be achieved, using optimized nanoparticles and electroporation. So far SLNs, used in our study, have been the largest nanocapsules whose transport increased due to the application of electroporation. This enhancement can be effectively applied in drug delivery, especially in the case when higher active drug volume is required.

## Electronic supplementary material

Below is the link to the electronic supplementary material.

Supplementary material 1 (DOCX 14333 kb)
