# Supplementary material for: The Effect of Millisecond Pulsed Electric Fields (msPEF) on Intracellular Drug Transport with Negatively Charged Large Nanocarriers Made of Solid Lipid Nanoparticles (SLN): In Vitro Study
Source: J Membr Biol. 2016 May 12;249(5):645–61. doi: 10.1007/s00232-016-9906-1 (PMC5045845; doi:10.1007/s00232-016-9906-1)
Supplement: Supplementary file 1 — Supplementary material 1 (DOCX 14333 kb) [file 232_2016_9906_MOESM1_ESM.docx]

**Supplemental Information (SI)**

**
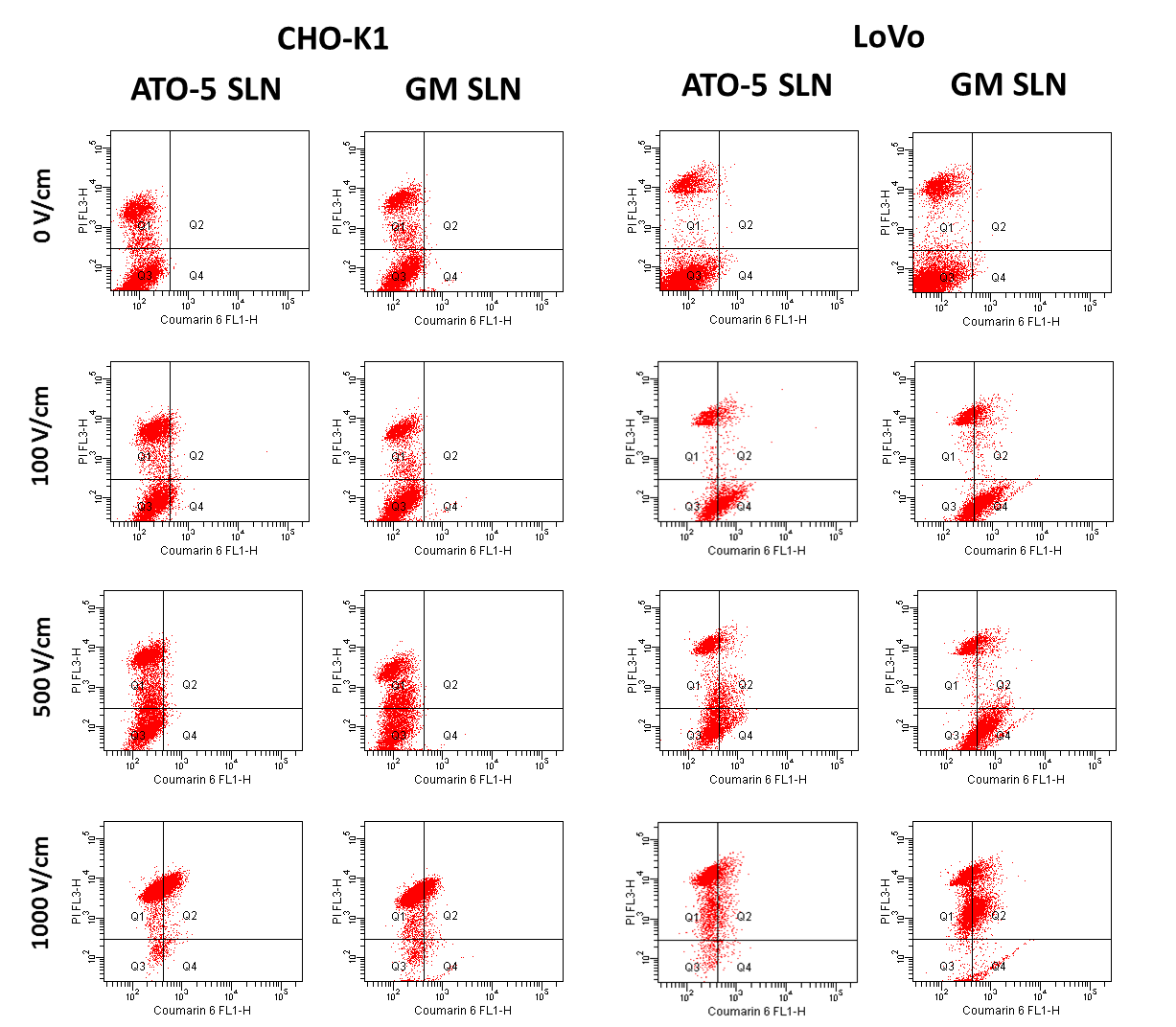
**

**Fig. SI-1.** FACS analysis for CHO-K1 and LoVo cells electroporated with PI and treated SLNs (dot plots).

**
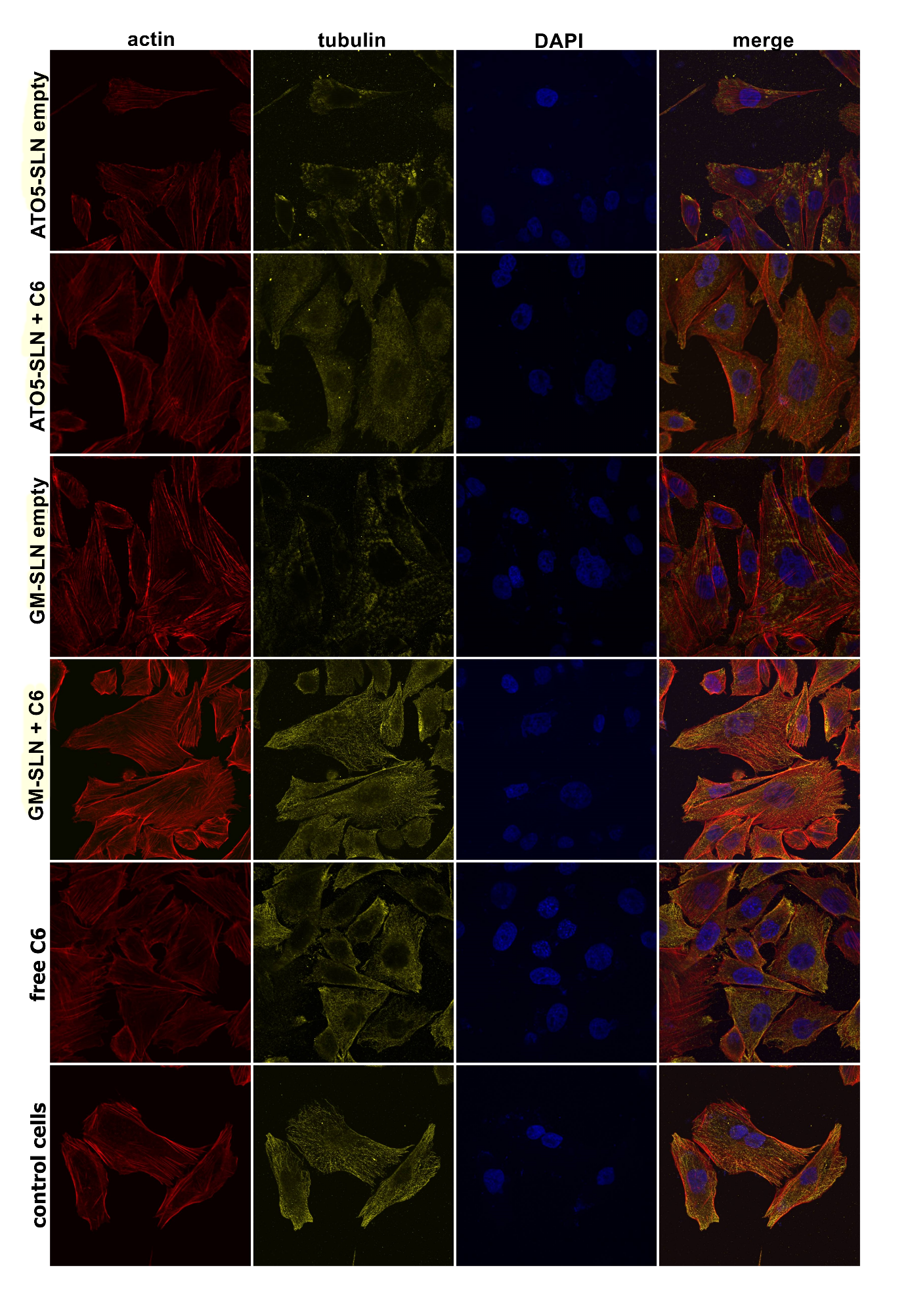
**

**A**

**
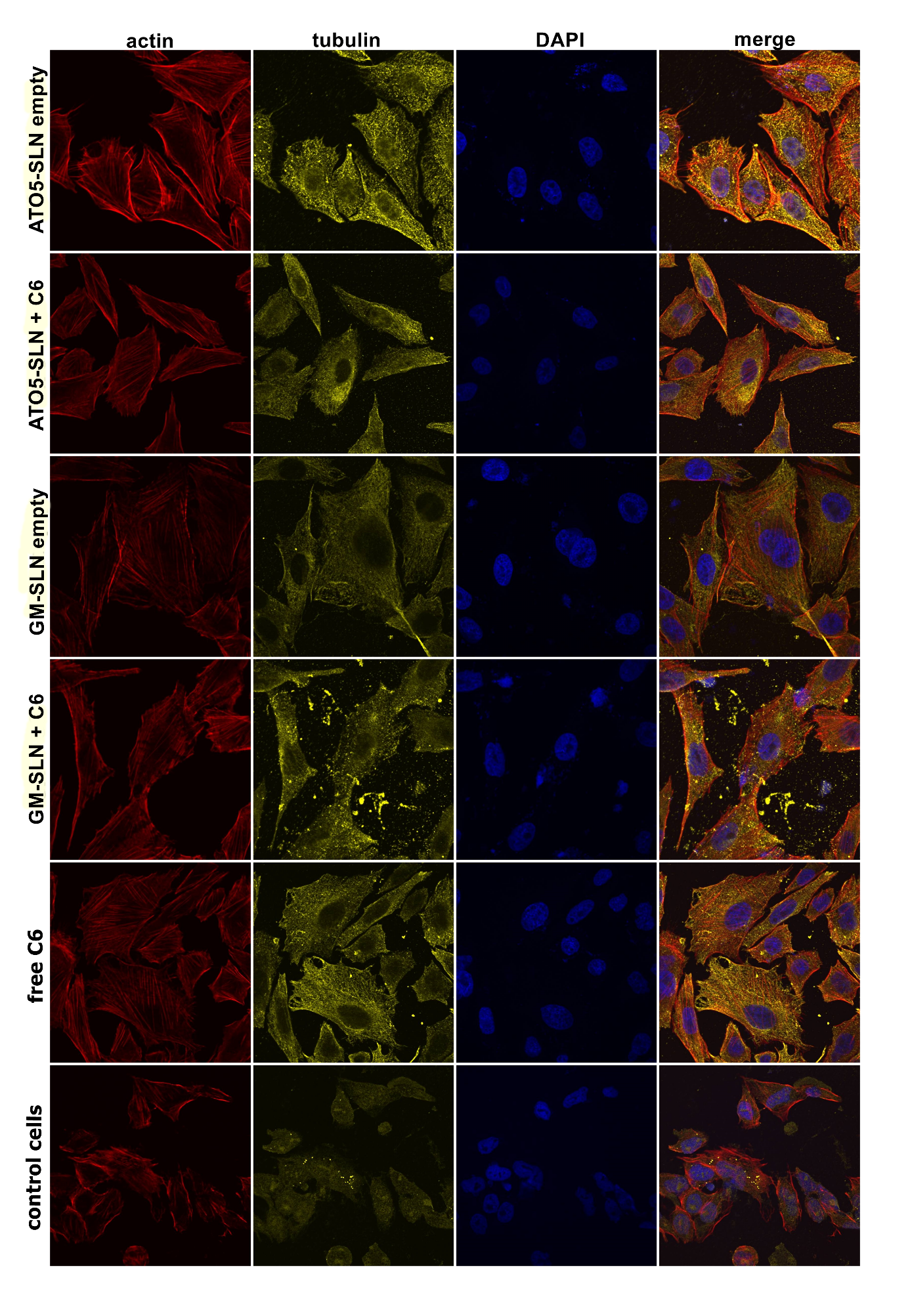
**

**B**

**Fig.SI-2.** The evaluation of cytoskeleton proteins in CHO-K1 cells: **a)** after incubation with coumarin 6 (C6)-loaded solid lipid nanoparticles - GM-SLN and ATO5-SLN; **b**) after treatment with SLNs supported with electroporation.

**
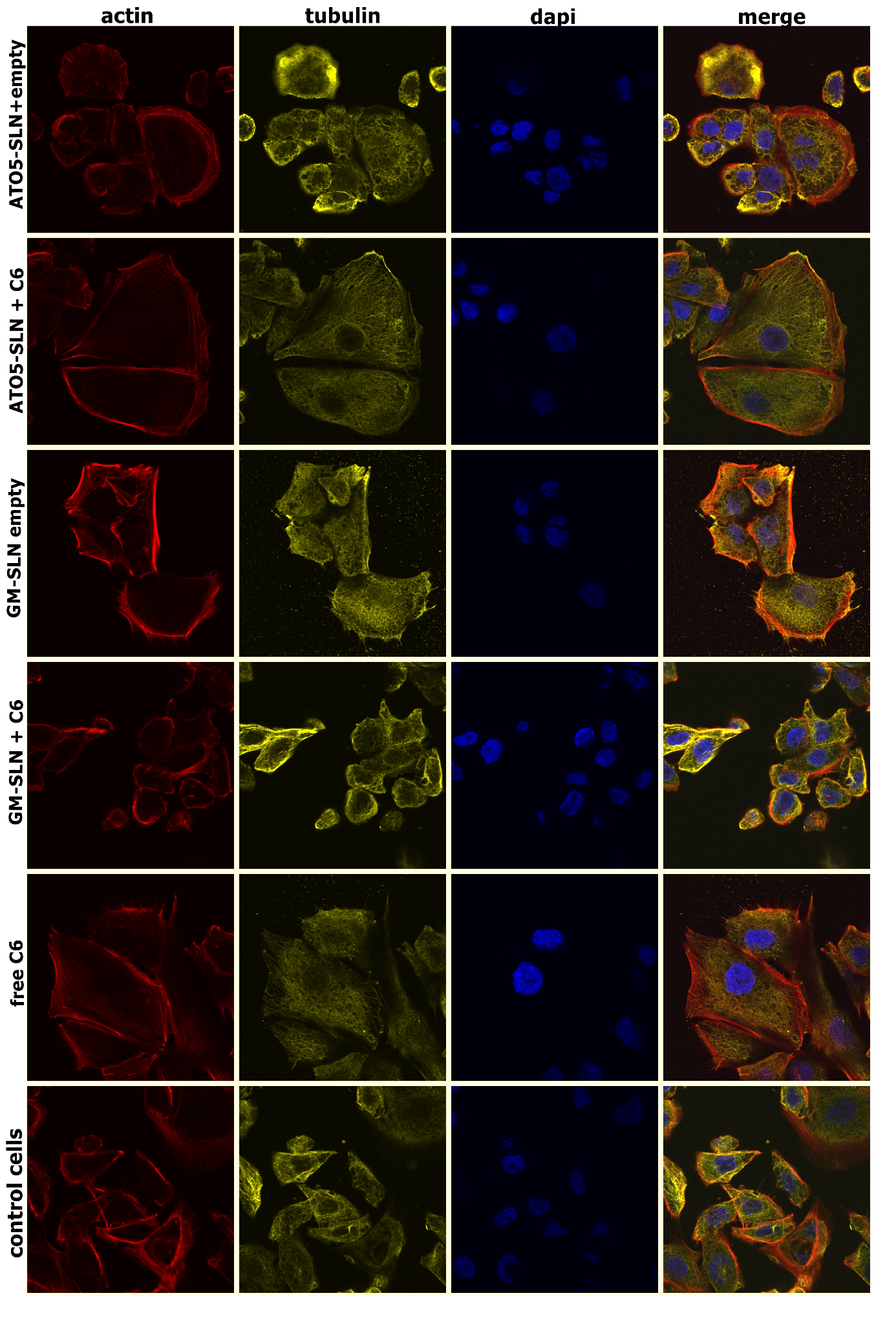
**

**A**

**
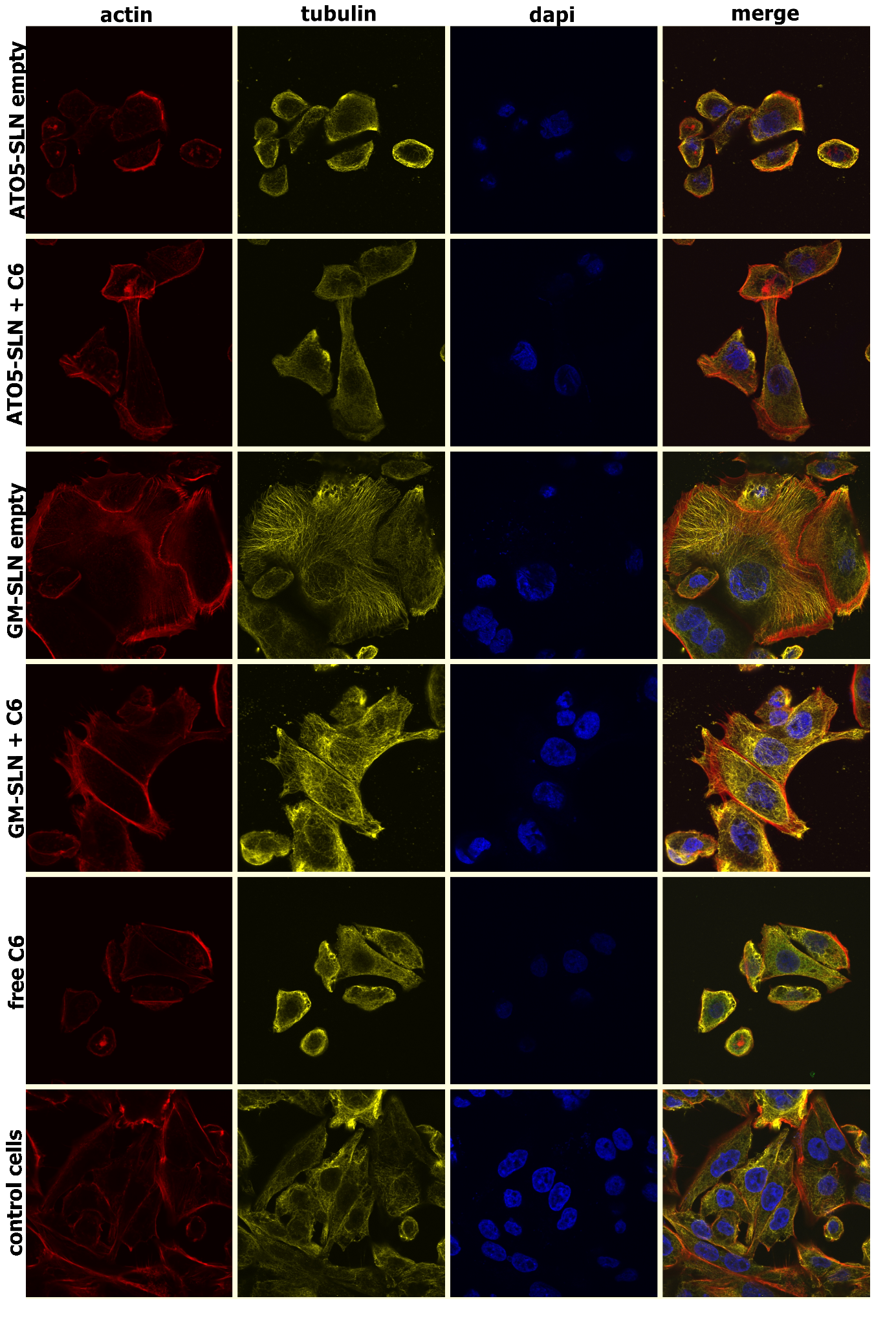
**

**B**

**Fig. SI-3.** The evaluation of cytoskeleton proteins in LoVo cells **a**) after incubation with coumarin 6 (C6)-loaded solid lipid nanoparticles - GM-SLN and ATO5-SLN; **b**) after treatment with SLNs supported with electroporation.
